# Esophageal squamous cell carcinoma invasion is inhibited by Activin A in ACVRIB-positive cells

**DOI:** 10.1186/s12885-016-2920-y

**Published:** 2016-11-09

**Authors:** Holli A. Loomans, Shanna A. Arnold, Laura L. Quast, Claudia D. Andl

**Affiliations:** 1 Department of Cancer Biology, Vanderbilt University, Nashville, TN USA; 2 Department of Veterans Affairs, Tennessee Valley Healthcare System, Nashville, TN USA; 3 Department of Pathology, Microbiology and Immunology, Vanderbilt University Medical Center, Nashville, TN USA; 4 Department of Surgery, Vanderbilt University Medical Center, Nashville, TN USA; 5 Burnett School of Biomedical Sciences, College of Medicine, University of Central Florida, 4110 Libra Drive, Building 20, BMS 223, Orlando, FL 32816 USA

**Keywords:** Dysplasia, Esophageal cell invasion, Cell signaling, Fibronectin, Podoplanin, Angiogenesis

## Abstract

**Background:**

Esophageal squamous cell carcinoma (ESCC) is a global public health issue, as it is the eighth most common cancer worldwide. The mechanisms behind ESCC invasion and progression are still poorly understood, and warrant further investigation into these processes and their drivers. In recent years, the ligand Activin A has been implicated as a player in the progression of a number of cancers. The objective of this study was to investigate the role of Activin A signaling in ESCC.

**Methods:**

To investigate the role Activin A plays in ESCC biology, tissue microarrays containing 200 cores from 120 ESCC patients were analyzed upon immunofluorescence staining. We utilized three-dimensional organotypic reconstruct cultures of dysplastic and esophageal squamous tumor cells lines, in the context of fibroblast-secreted Activin A, to identify the effects of Activin A on cell invasion and determine protein expression and localization in epithelial and stromal compartments by immunofluorescence. To identify the functional consequences of stromal-derived Activin A on angiogenesis, we performed endothelial tube formation assays.

**Results:**

Analysis of ESCC patient samples indicated that patients with high stromal Activin A expression had low epithelial ACVRIB, the Activin type I receptor. We found that overexpression of stromal-derived Activin A inhibited invasion of esophageal dysplastic squamous cells, ECdnT, and TE-2 ESCC cells, both positive for ACVRIB. This inhibition was accompanied by a decrease in expression of the extracellular matrix (ECM) protein fibronectin and podoplanin, which is often expressed at the leading edge during invasion. Endothelial tube formation was disrupted in the presence of conditioned media from fibroblasts overexpressing Activin A. Interestingly, ACVRIB-negative TE-11 cells did not show the prior observed effects in the context of Activin A overexpression, indicating a dependence on the presence of ACVRIB.

**Conclusions:**

We describe the first observation of an inhibitory role for Activin A in ESCC progression that is dependent on the expression of ACVRIB.

**Electronic supplementary material:**

The online version of this article (doi:10.1186/s12885-016-2920-y) contains supplementary material, which is available to authorized users.

## Background

Esophageal cancer is the eighth most prevalent cancer and sixth most common cause of cancer-related deaths globally [1, 2]. The different subtypes are esophageal adenocarcinoma (EAC) and esophageal squamous cell carcinoma (ESCC). Though the prevalence of EAC has now surpassed that of ESCC in North America and Europe, ESCC remains the dominant subtype globally (~80 %), with the highest incidence and mortality occurring in developing countries in Asia [2–4]. ESCC poses a great public health challenge, as little progress has been made in improving diagnosis and outcomes for patients. Approximately 80 % of ESCC is diagnosed in late stage and has only a 15 % 5-year survival rate; these statistics have remained stagnant over the last 20 years [1–3]. Though the list of targeted therapies is constantly growing, therapeutic resistance and recurrence persist to occur [2–5]. To address this problem, an examination of new diagnostic and prognostic indicators, as well as the mechanisms underlying progression, is needed to provide further insight into new methods to combat ESCC.

Activin A, a homodimer of inhibin β_A_ subunits, has been indicated as a key player in ovarian, prostate and breast cancers [6, 7]. A member of the TGFβ superfamily, Activin A, upon binding an Activin type II (ActRII/B) and type I (ACVRI/B) receptor, subsequently activates the Smad cascade thereby driving transcription of target genes (reviewed in [8]). Activin A was initially discovered as a gonadotroph, where it acts as a potent inducer of cell cycle arrest [9, 10]. In vivo knock-down of inhibin β_A_ in mice leads to incomplete development [11] and defects in squamous tissue wound healing [12]. Although previously linked to carcinogenesis [13], the mechanism and precise contribution of Activin A to initiation and progression remains to be elucidated. Studies in gastric cancer have shown Activin A to be a potent inhibitor of angiogenesis and inducer of apoptosis [14, 15]; however, similar to TGFβ, Activin A can act as a tumor promoter or suppressor in different contexts. In breast cancer, some reports have indicated that Activin A can induce cell growth and epithelial-mesenchymal transition [16, 17], whereas other studies have demonstrated that Activin A treatment induces cell cycle arrest and inhibits growth [10, 18]. This “dual role” phenomenon has also been observed in prostate [19] and lung [20, 21] cancers.

In the esophagus, clinical and experimental evidence has indicated that Activin A promotes cancer progression. Clinical studies have correlated increased Activin A expression with tumor aggressiveness, differentiation status [22], and poor patient prognosis [23]. Several explanations have been suggested to explain the mechanisms induced by Activin A. In ESCC, one such proposal for Activin A contribution to tumor progression is through the induction of N-cadherin, with subsequent loss of E-cadherin, a feature that has been associated with increased tumor aggressiveness [24]. Additional evidence has suggested that Activin A can upregulate MMP-7 expression, which has been correlated with gastric and colorectal cancers [25] via the transcription factor AP-1, a non-canonical pathway [26].

Taken together, contrary to its characterization as an inhibitor of angiogenesis and growth suppressor, Activin A overexpression contributes to ESCC indicating that during cancer progression this signaling pathway switches function from an anti-tumorigenic to pro-tumorigenic regulation. Of particular importance is the contribution of the microenvironment in this context, which we investigate here. Activin A has been shown to not only exert functional effects on tumor cells, but also on stromal cells located within the microenvironment, where Activin A can induce a “wound healing” phenotype (reviewed in [27]).

To address the contrasting roles of Activin A, a growth inhibitor known to be highly expressed in several cancers, we aimed to determine the source of Activin A (stromal versus epithelial-secreted) and to mimic stromal overexpression in an organotypic reconstruct culture system. In this study, we show that, in a dysplastic esophageal microenvironment, fibroblast-secreted Activin A, as one source of stromal Activin A, suppresses cell invasion through the inhibition of epithelial cell proliferation and the regulation extracellular matrix components. We further observed that esophageal TE-2 tumor cells respond to fibroblast-derived Activin A with inhibition of cell invasion similar to the dysplastic cells, yet this effect was not observed in the Activin receptor IB (ACVRIB)-negative ESCC cell line TE-11. We conclude that during cancer progression, in concordance with upregulated stromal Activin A expression, ACVRIB is frequently downregulated in ESCC cells, allowing an escape from the inhibitory effects of Activin A as a novel mechanism of esophageal tumorigenesis.

## Methods

### Cell lines and cell culture

Fetal esophageal fibroblasts were cultured in Dulbecco’s Modified Eagle Medium (DMEM) (Gibco, Grand Island, NY) supplemented with 10 % fetal bovine serum (FBS) (Atlanta Biologicals, Norcross, GA) and 1 % penicillin and streptomycin (P/S) (Gibco) [28]. Primary esophageal keratinocytes expressing dominant-negative mutants of E-cadherin and TβRII (ECdnT), established as previously described [29], were cultured in keratinocyte serum-free media (KSFM) supplemented with 40 μg/mL bovine pituitary extract, 1 ng/mL epidermal growth factor (EGF), and 1 % P/S (Gibco). The ESCC cell lines TE-2 and TE-11 were grown in RPMI and DMEM (Gibco), respectively, and supplemented with 10 % FBS and 1 % P/S [30]. The endothelial cell line HMEC-1 were cultured in MCDB131 (Gibco) supplemented with 10 % FBS, 10 ng/ml EGF, 1 μg/ml hydrocortisone (Sigma), and 1 % P/S [31].

### Retrovirus infection

Overexpression of Activin A was performed using a retroviral construct containing cDNA for *INHBA*, the sequence that encodes the inhibin β_A_ subunit, as previously described [32]. The vector backbone pBABE-zeo was purchased from AddGene (plasmid #1766 [33]) and inhibin β_A_ cDNA was inserted at the multiple cloning site. Virus was generated using Phoenix-Ampho HEK293T cells (ATCC CRL-3213). Fibroblasts (Fibro-ActA) were then transduced and selected using Zeocin (Life Technologies) at a concentration of 800 μg/ml. Fibroblasts transduced with an empty pBABE-zeo vector (Empty) and untransfected parent fibroblasts (Parent) were used as controls. Activin A overexpression was validated by ELISA.

### Cell contraction assay

Cell contraction assay was performed according to the manufacturers’ protocol (Cell Biolabs, Inc., San Diego, CA). 3-butanedione monoxime (BDM) was used as a control.

### ELISA

Capture ELISAs for Activin A were purchased from R&D Systems (Minneapolis, MN) and performed using conditioned media according to the manufacturers’ instructions.

### Organotypic culture

Organotypic reconstruct cultures were performed as previously described [34]. Briefly, parental, empty, or fibroblasts with Activin A overexpression were seeded into a 3D matrix (75,000 cells/well) containing collagen I and Matrigel (BD Biosciences, Franklin Lakes, NJ) and allowed to incubate for 7 days at 37 °C. Following incubation, ECdnT, TE-2, or TE-11 cells were seeded on top of the fibroblast matrix (500,000 cells/well). Cultures were then allowed to incubate an additional 10 days. Treatments were added to the cultures beginning two hours following epithelial cell seeding and refreshed every 2 days. A neutralizing antibody against Activin A (nAb; R&D Systems) and A83-01, a chemical inhibitor of TGFβ/Activin A/BMP type I receptors (ACVRIB/TβRI/ALK7) (Tocris, Bristol, UK), were used for treatment.

### Staining

#### Immunofluorescence of FFPE sections

At time of harvest, organotypic cultures were fixed in 10 % formalin and embedded in paraffin. Cultures were cut to 5 μm sections, deparaffinized, and heated for 12 min in 1XTE buffer in a pressure cooker to perform antigen retrieval. Sections were blocked with 1XPBS containing 5 % bovine serum albumin (1XPBST + BSA; Sigma-Aldrich) for one hour at room temperature. The sections were incubated with primary antibody diluted in 1XPBS + BSA overnight at 4 °C. The following day, sections were washed three times with 1XPBS, and incubated with a secondary antibody with a conjugated fluorophore (anti-rabbit Texas Red 1:200, Vector Laboratories, Burlingame, CA; anti-mouse DyLight Alexa488 1:200, Vector Laboratories; anti-rat Alexa594 1:200, Life Technologies), diluted in 1XPBS + BSA, for 1–2 h at room temperature. Sections were washed three times and mounted using ProLong Gold anti-fade with DAPI (Life Technologies). Sections were imaged on a Zeiss microscope, using Axiocam and AxioVision software (Carl Zeiss Microscopy, Thornwood, NY). Antibodies used for immunofluorescence are listed in Table 1.

**Table 1:** Antibodies used for immunofluorescence

Antibody	Dilution	Application	Vendor
Podoplanin	1:500	Immunofluorescence	eBioscience
Vimentin	1:1000	Immunofluorescence	Sigma
N-cadherin	1:500	Immunofluorescence	BD Bioscience
Fibronectin	1:1000	Immunofluorescence	BD Bioscience
E-cadherin	1:1000	Immunofluorescence	BD Bioscience
Vimentin	1:50	Immunofluorescence	Cell Signal Technology
Laminin 5γ2	1:200	Immunofluorescence	Santa Cruz Biotechnology
Ki67	1:200	Immunofluorescence	Cell Signal Technology
Collagen IV	1:200	Immunofluorescence	Cell Signal Technology
αSMA	1:200	Immunofluorescence	Sigma
COL4A2	1:100	TMA	Chondrex
INHBA	1:200	TMA	ProteinTech
CD68	1:20	TMA	R&D Systems
ACVRIB	1:200	TMA	Abcam
Keratin 14	1:50	TMA	Abcam

### Endothelial tube formation assay

Growth factor-reduced Matrigel (Corning) was added to each well of a 96-well plate and allowed to solidify at 37 °C for approximately 30 min. HMEC-1 cells, in the appropriate conditioned media treatment, were seeded at 15,000 cells/well, in triplicate, to the Matrigel-containing wells and incubated for 18 h at 37 °C. Following incubation, bright field images of each well were taken and analyzed using Angiogenesis Analyzer for ImageJ [35]. This software allows network organization analysis of a skeleton or tree, extremities or nodes and junctions in a binary image (http://image.bio.methods.free.fr/ImageJ/?Angiogenesis-Analyzer-for-ImageJ&lang=en&artpage=3-7#outil_sommaire_3). Statistical analysis was performed by one-way ANOVA in GraphPad.

### ESCC tissue microarray

Microarrays were purchased from US Biomax (Rockville, MD). Following immunofluorescence staining, cores were quantified using the “Measure Stained Area Fluorescence” algorithm as part of the Leica Microsystems Tissue IA version 4.0.6 program (Buffalo Grove, IL). Fluorescence area was measured in μm^2^. Antibodies used for immunofluorescence are listed in Table 1.

### Statistical analysis

Experimental results were analyzed using Student’s t-test or one-way ANOVA and expressed as the mean +/- standard deviation. Statistical analysis of the in vitro experiments was performed in Prism 6.0 (GraphPad, San Diego, CA). ESCC microarrays were analyzed using linear generalized estimating equations (GEE) or Kruskal-Wallis tests. For matched samples, Wilcoxon signed rank tests were used. Results are expressed as the mean +/- standard deviation. Statistical analysis of the microarrays was performed using SPSS (IBM, Armonk, NY).

## Results

### Epithelial ACVRIB expression levels are dependent upon expression of stromal Activin A in ESCC

**Fig. 1:**
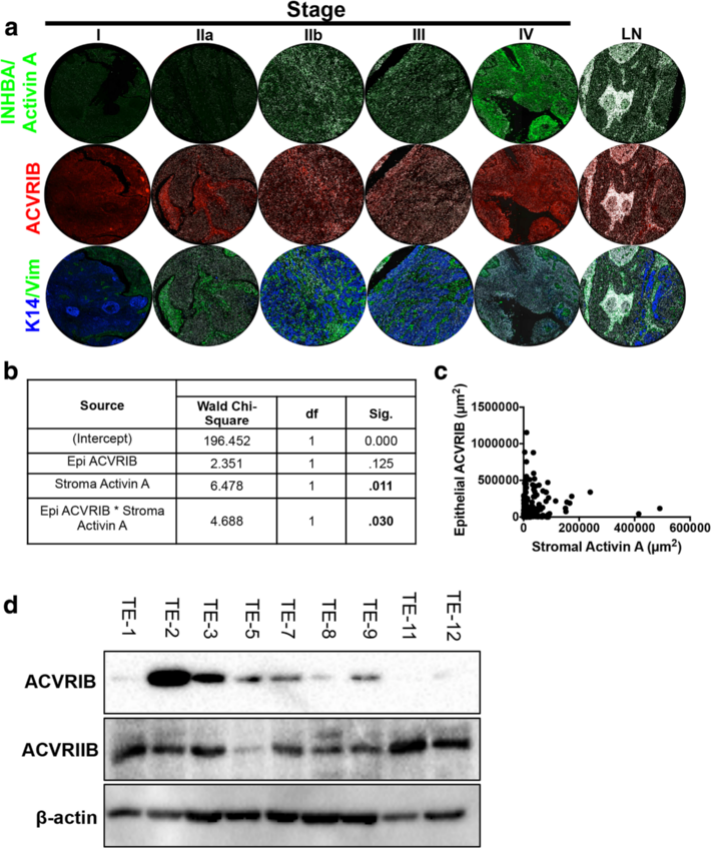
Epithelial ACVRIB expression in ESCC patient samples is dependent upon the expression of Activin A. **a** Representative immunofluorescence staining of microarray samples (whole section image shown of tumor tissues Stage I-IV and lymph node [LN]) for INHBA/Activin A, ACVRIB, K14, and vimentin (Vim). **b** Using a linear generalized estimating equation (GEE), a significant interaction was found between total Activin A expression and epithelial ACVRIB as a predictor of stage. Though epithelial ACVRIB expression was not a predictor of stage alone, we found that stromal INHBA/Activin A and epithelial ACVRIB expression showed a significant interaction. Analysis of immunofluorescence was performed on the whole tissue section. (TMA = 200 cores) **c** Graphical illustration of the relationship between epithelial expression of ACVRIB and stromal expression of ACVRIIB, measured by intensity in μm^2^, in the TMA samples. Each dot represents a single patient sample. **d** Western blot of ACVRIB and ACVRIIB of the TE series of ESCC cell lines. β-actin was used as a loading control

It has been well established that Activin A expression in normal physiology is low with increased expression in invasive cancer [23, 36–38]. We first aimed to determine the localization and expression level of Activin A in ESCC tissues. Using commercially purchased tissue microarrays, we stained 200 esophageal tissues (cancer adjacent, squamous cell carcinoma, and lymph node [LN] metastases) from 120 patients for Activin A and ACVRIB, the primary type I receptor involved in Activin A signaling (Fig. 1a). Additional staining for keratin 14 (K14) to determine squamous cells, vimentin (Vim) to identify mesenchymal cells, and collagen as well as CD68, a glycoprotein expressed in monocytes, were used as controls. These markers allowed us to compartmentalize the localization of Activin A and ACVRIB localization to epithelial/tumor or stroma. Data are quantified in Additional file 1: Figure S1. Using Kruskal-Wallis tests and generalized estimating equations (GEE), we found that Activin A expression alone was not associated with tumor stage in this set of tumor tissues, similarly ACVRIB expression alone varied between stage and was not a predictor of tumor stage (data not shown). However, we identified that after controlling for epithelial ACVRIB expression, Activin A expression in the stroma was, indeed, a significant predictor of stage, when analyzed by multivariable GEE analysis (Fig. 1b). Therefore, we determined that with increasing stromal Activin A expression, epithelial ACVRIB expression decreases within this patient data set, thereby, promoting a more aggressive ESCC phenotype as illustrated by increased stage. This relationship is illustrated in Fig. 1c. We next examined 9 commonly utilized ESCC cell lines for protein expression of ACVRIB and ACVRIIB, the primary Activin A type II receptor (Fig. 1d). ACVRIB expression was low in 3 out of 9 cell lines and barely detectable in 4 out of 9, indicating decreased expression in 7 out of 9 ESCC cell lines. On the contrary, ACVRIIB was downregulated in only one cell line, TE-5. To reconcile the high Activin A levels in the context of decreased ACVRIB expression and the functional consequences, we next grew dysplastic and TE-2 and TE-11 cancer cell lines in organotypic cultures.

### Fibroblast-secreted Activin A inhibits cell invasion of dysplastic esophageal cells and regulates extracellular matrix protein expression

Fibroblasts play a substantial role in inflammation, wound healing, and extracellular matrix (ECM) deposition [39, 40]. In the context of cancer, fibroblasts are recruited by cancer cells via epithelial-mesenchymal crosstalk to rearrange the ECM to create a ‘reactive’ stroma, which provides an environment conducive to cell invasion [41, 42]. Microenvironment-derived Activin A has been implicated as part of the reactive stroma phenotype [43, 44]. Considering elevated levels of Activin A are found in the stroma despite the characteristic function of Activin A as an inducer of growth arrest, we first aimed to investigate the role of microenvironment-derived Activin A in a three-dimensional organotypic reconstruct (OTC) model in the presence of dysplastic esophageal keratinocytes (ECdnT) [29]. As normal esophageal fibroblasts secrete low to negligible levels of Activin A ([34] and data not shown), we retroviral transduced fibroblasts with *INHBA* to achieve Activin A overexpression levels similar to those observed in cancer-associated fibroblasts [34, 43]. Upon embedding Activin A overexpressing fibroblasts (Fibro-ActA) in the organotypic culture matrix, we validated overexpression and secretion of Activin A by ELISA (Additional file 2: Figure S2a). Fibro-ActA secreted significantly more Activin A than the tested epithelial cells, ensuring that the majority of Activin A in OTC would be derived from the fibroblasts. To confirm that Activin A overexpression was maintained during the course of each OTC (17 days), we collected media every 2 days and measured Activin A concentrations by ELISA (Additional file 2: Figure S2b-d). Parent and empty vector fibroblasts were used as controls.

**Fig. 2:**
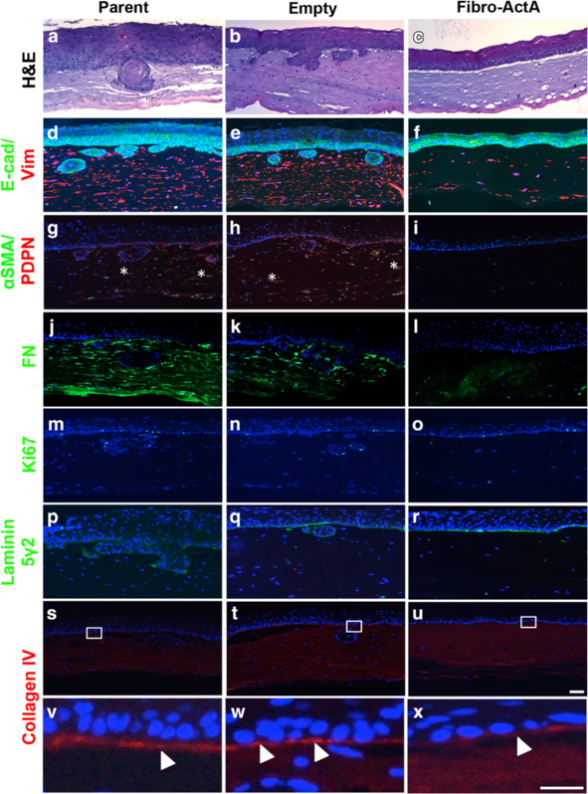
Overexpression of Activin A in the dysplastic esophageal microenvironment inhibits extracellular matrix protein reorganization. **a**-**c** Hematoxylin and eosin staining of parent, empty, and Fibro-ActA organotypic cultures. **d**-**f** Three-dimensional organotypic Fibro-ActA cultures exhibit no alterations in epithelial ECdnT E-cadherin (E-cad) expression, however vimentin (Vim) is downregulated in the fibroblasts, as examined by immunofluorescence. **g**-**i** αSMA expression was substantially downregulated in the fibroblasts, while podoplanin (PDPN) expression was downregulated in both epithelial cells and fibroblasts. The asterisks(*) in the parental and empty vector cultures denote co-localization of αSMA and PDPN, a common characteristic of cancer-associated fibroblasts. **j**-**l** Fibronectin (FN) deposition was decreased in Fibro-ActA cultures compared to parent and empty vector controls. **m**-**r** Ki67, a marker of proliferation, and laminin 5γ2, a marker of basal cell differentiation, was unchanged between conditions. **s**-**u** Collagen IV deposition, a primary component of basement membrane deposition, was decreased in Fibro-ActA compared to control. **v**-**x** Collagen IV staining, higher magnification of boxed region in s-u. Arrows indicate the collagen IV basement membrane, laid by the epithelial cells. (Short scale bar = 20 μm; long scale bar = 5 μm) (*n* = 4)

ECdnT cells showed collective cell invasion and keratin pearl formation characteristic of an invasive ESCC when cultured with control parent and empty vector fibroblasts (Fig. 2a, b). When cultured with Fibro-ActA, ECdnT cell invasion was suppressed (Fig. 2c). Immunofluorescence staining was performed with anti-E-cadherin (E-cad) antibody to identify the epithelial compartment. An examination of fibroblast protein expression by immunofluorescence showed that vimentin (Vim), a mesenchymal marker, andαSMA and podoplanin (PDPN), markers of fibroblast differentiation and activation, were downregulated in Fibro-ActA cultures (Fig. 2d-i). We also observed substantial downregulation of the ECM protein fibronectin (FN) (Fig. 2j-l). Interestingly, the ability of Fibro-ActA to interact with and contract the ECM was not altered until the epithelial cells were seeded (Additional file 2: Figure S2e, f), indicating the necessity of epithelial-mesenchymal crosstalk to modify contractility. Epithelial cell proliferation, measured by Ki67 staining, did not change between conditions (Fig. 2m-o, Additional file 3: Figure S3a). Interestingly, in all conditions, epithelial cells deposited laminin 5γ2, a squamous epithelium basement membrane marker [45], and collagen IV, a major basement membrane component (Fig. 2p-r) [46]. Collagen IV localization to the basement membrane, however, was slightly reduced in Fibro-ActA cultures (Fig. 2s-x, arrows). These results support the role of Activin A as an invasion suppressor and indicate Activin A-dependent regulation of ECM-associated proteins.

### Inhibition of Activin A signaling during dysplasia restores extracellular matrix protein expression

**Fig. 3:**
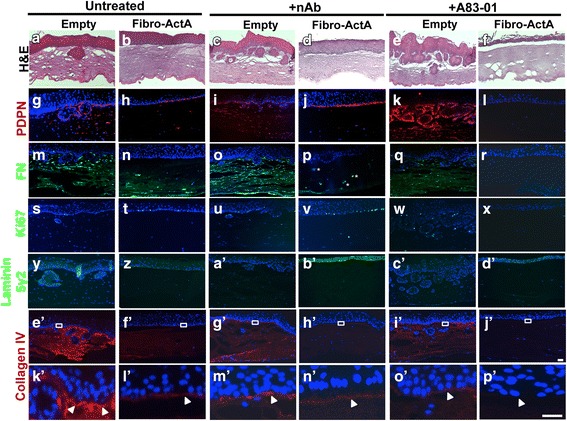
Inhibition of the Activin A ligand, but not the receptor, restores extracellular matrix protein expression. (**a**-**f**) Hematoxylin and eosin staining shows that ECdnT cells didn’t invade in any of the Fibro-ActA cultures, however invasion was increased in empty vector cultures treated with an Activin A neutralizing antibody (nAb) or A83-01. (**g**-**l**) Epithelial cell podoplanin (PDPN) expression was restored to control levels in Fibro-ActA cultures following treatment with nAb. (**m**-**r**) Treatment of Fibro-ActA cultures with nAb partially restored stromal fibrillar fibronectin (FN) expression (asterisks) to untreated empty vector control levels. This effect was not observed following treatment with A83-01. (**s**-**x**) Laminin 5γ2 expression was increased in Fibro-ActA cultures treated with nAb and A83-01. (**y**-**d’**) Ki67 staining remained relatively unchanged between conditions. (**e’**-**j’**) Collagen IV deposition at the basement membrane was restored to the expression level of the control upon treatment of Fibro-ActA with nAb. This deposition was not observed in Fibro-ActA cultures treated with A83-01. (**k’**-**p’**) Collagen IV staining, higher magnification of boxed region in **e’**-**j’**. Arrows indicate the collagen IV basement membrane, laid by the epithelial cells. (Short scale bar = 20 μm; long scale bar = 5 μm) (*n* = 2)

To demonstrate Activin A-dependence and specificity of epithelial invasion inhibition and the expression of several ECM proteins, we used two separate approaches for Activin A inhibition: a neutralizing antibody specific for the Activin A ligand (nAb) and A83-01, a chemical inhibitor of TGFβ/Activin A/BMP type I receptors (ACVRIB/TβRI/ALK7) [47]. We have previously shown the ability of nAb and A83-01 to neutralize Activin A signaling in this model system [34, 48]. Treatment with nAb increased cell invasion in dysplastic empty vector control cells, yet could not overcome the inhibition of cell invasion in the context of Fibro-ActA cultures (Fig. 3a-d). Similarly, A83-01 treatment, while increasing cell invasion in the ECdnT cultures with empty vector control, could not restore cell invasion in the Fibro-ActA cultures (Fig. 3e, f). When we examined ECM protein expression in these cultures by immunofluorescence, treatment with nAb could restore expression of fibrillar fibronectin in Fibro-ActA, similar to that observed in empty vector untreated cultures, yet A83-01 prevented their restoration. We also observed restoration of podoplanin (Fig. 3g-r). This indicates the necessity of Activin A signaling to induce expression of these proteins. Laminin 5γ2 was upregulated in the Fibro-ActA cultures treated with both nAb and A83-01, the only tested marker to do so (Fig. 3s-x). This result indicates that one potential mechanism of regulating laminin 5γ2 expression is through the Activin A-ACVRIB axis. Epithelial cell proliferation, measured by Ki67, was inhibited with the addition of A83-01 to all cultures, but was unaltered in the presence of nAb (Fig. 3y-d’). Interestingly, when we examined the basal epithelial layer of the Fibro-ActA cultures treated with nAb, we also found collagen IV deposition at the basement membrane (Fig. 3e’-h’). This was not observed in cultures treated with A83-01 (Fig. 3i’-j’; higher magnification Fig. 3k’-p’). We, therefore, conclude that the blocking of Activin A-receptor binding is necessary to inhibit the observed ECM alterations, yet not sufficient to induce cell invasion.

### Stromal Activin A inhibits TE-2 cell invasion in three-dimensional culture

**Fig. 4:**
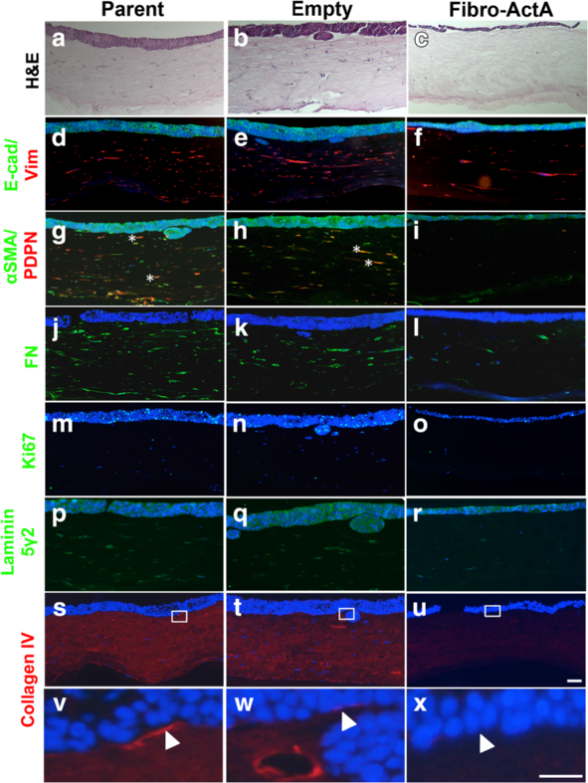
Overexpression of Activin A shows similar extracellular matrix protein regulation in ACVRIB-expressing ESCC. **a**-**c** Hematoxylin and eosin staining of parent, empty, and Fibro-ActA organotypic cultures, cultured with TE-2 ESCC cells. **d**-**f** Three-dimensional organotypic Fibro-ActA cultures exhibit no alterations in epithelial ECdnT E-cadherin (E-cad) expression, however vimentin (Vim) is downregulated in the fibroblasts, as examined by immunofluorescence. **g**-**i** αSMA and podoplanin (PDPN) expression was significantly downregulated in the fibroblasts and epithelial cells, respectively. The asterisks(*) in the parental and empty vector cultures denote co-localization. **j**-**l** Fibronectin (FN) deposition was decreased in Fibro-ActA cultures compared to parent and empty vector controls. **m**-**o** Ki67 staining was decreased in epithelial TE-2 cells cultured with Fibro-ActA, compared to parent and empty vector cultures (quantification in Additional file 3: Figure S3). **p**-**r** Laminin 5γ2, a marker of basal cell differentiation, was unchanged between conditions. **s**-**u** Collagen IV deposition, a primary component of basement membrane deposition, was decreased in Fibro-ActA compared to control. **v**-**x** Collagen IV staining, higher magnification of boxed region in s-u. Arrows indicate the collagen IV basement membrane, laid by the epithelial cells. (Short scale bar = 20 μm; long scale bar = 5 μm) (*n* = 2)

As we observed that fibroblast-derived overexpression of Activin A inhibited cell invasion in premalignant cells, we aimed to investigate if Activin A has a similar effect on cancer cells. We cultured the ESCC cell line TE-2 with Fibro-ActA. TE-2 cells express the Activin A receptor complex components, ACVRIIB and ACVRIB (Fig. 1d). Similar to the dysplastic model, TE-2 cells were unable to invade into the stromal layer when cultured with Fibro-ActA, compared to controls (Fig. 4a-c). As in the ECdnT cultures (Fig. 2), E-cadherin marks the epithelial layer and vimentin labels the fibroblasts in the stromal compartment (Fig. 4d-f). Interestingly, we observed co-localization of αSMA and podoplanin in the control fibroblasts (Fig. 4g-h, asterisks), not seen in the Fibro-ActA fibroblasts, suggesting differentiation to a myofibroblast lineage in the invasive tumor cultures [49].

Unlike the ECdnT cultures, we observed differences in Ki67 staining between TE-2 Fibro-ActA and control cultures indicating significantly decreased proliferation compared to controls, as indicated by Ki67 staining (Fig. 4m-o, Additional file 3: Figure S3b). Similarly to our observations with in the premalignant ECdnT model, laminin 5γ2 expression was unchanged between conditions (Fig. 4p-r). Collagen IV deposition in the basal layer was downregulated in Fibro-ActA (Fig. 3s-x).

Overall, these observations suggest that the TE-2 ESCC cell line responds to the overexpression of Activin A by the fibroblasts in a similar manner to the dysplastic, premalignant ECdnT model. This prompted us to analyze the effects of Fibro-ActA on the TE-11 ESCC cell lines, which we determined has low ACVRIB protein expression (Fig. 1d).

### Cell invasion and regulation of the ECM proteins requires intact Activin A signaling

**Fig. 5:**
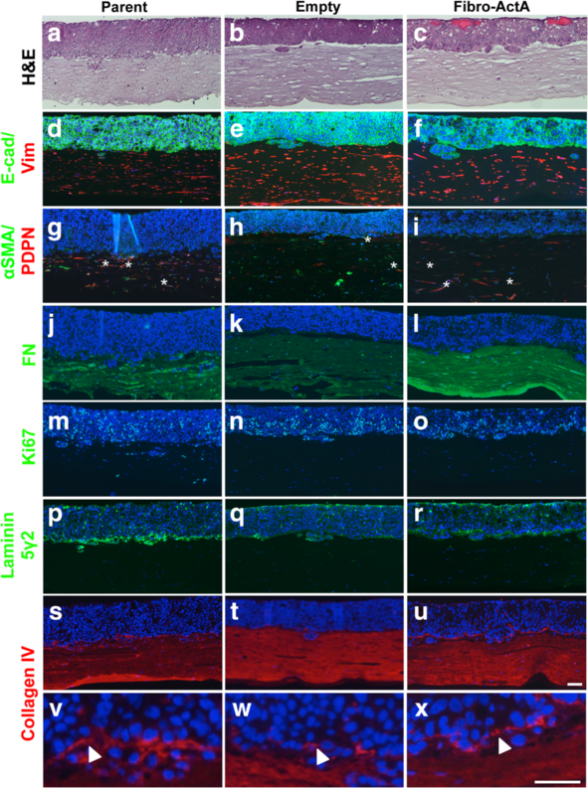
ACVRIB-negative ESCC shows epithelial alterations, however extracellular matrix protein expression remains unaltered. **a**-**c** Hematoxylin and eosin staining of parent, empty, and Fibro-ActA organotypic cultures, cultured with TE-11 ESCC cells. Fibro-ActA cultures show increased keratin deposition in the epithelial layer, compared to parent and empty vector controls. **d**-**f** Three-dimensional organotypic Fibro-ActA cultures exhibit no alterations in epithelial E-cadherin (E-cad) or stromal vimentin (Vim) expression, as examined by immunofluorescence. **g**-**i** αSMA and podoplanin (PDPN) expression was unchanged between conditions. Co-localization of the markers is denoted by asterisks(*). **j**-**l** Fibronectin (FN) deposition and (**m**-**o**) Ki67 and (**p**-**r**) laminin5γ2 expression was not different between control (parent and empty) and Fibro-ActA. **s**-**u** Collagen IV deposition, a primary component of basement membrane deposition, was decreased in Fibro-ActA compared to control. **v**-**x** Collagen IV staining, higher magnification of boxed region in s-u. Arrows indicate the collagen IV basement membrane, laid by the epithelial cells. (Short scale bar = 20 μm; long scale bar = 5 μm) (*n* = 2)

Cancer cells have the ability to adapt in response to environmental factors. One such mechanism of adaptation is cancer cell clonal expansion, where one cancer cell with a particular advantage is able to survive and expand [50]. Downregulation or loss of components of the TGFβ signaling cascade, such as TβRII, has been noted in several cancers and associated with increased aggressiveness [29, 51, 52]. Similar observations have been made regarding the members of the Activin A signaling cascade [53]. Given the observation that Activin A levels are high in the tumor setting and Activin A acts as an invasion suppressor for TE-2 cells, we used a ACVRIB-negative cell line, which has disrupted Activin A signaling due to the lost receptor complex component, to provide validation and mechanistic insight to the patient sample observations (Fig. 1). As ACVRIB is the primary signaling kinase for the Activin A cascade, as discussed above, we chose the cell line, TE-11, which had low ACVRIB expression (Fig. 1d) to evaluate the response of ACVRIB-negative cells to fibroblast-derived Activin A. TE-11 cells invaded into the stroma, shown by H&E staining, in both Fibro-ActA and control cultures (Fig. 5a-c). By immunofluorescence, we examined the same epithelial, stromal, and ECM markers used in the previous experiments (Figs. 2, 3 and 4). As expected, in the TE-11 cultures, Activin A secretion by the fibroblasts did not alter the overall expression of vimentin, αSMA, podoplanin, and fibronectin (Fig. 5d-l). Similarly to the TE-2 cultures, co-localization of αSMA and podoplanin was observed, however this was noted in all cultures, including Fibro-ActA (Fig. 5g-i, asterisks). Ki67 and laminin 5γ2 expression was unchanged between conditions (Fig. 5m-r). Deposition of collagen IV, a common characteristic of cancer cells, [54] was increased overall in TE-11 (Fig. 5s-x) [53]. In a related study analyzing Activin A signaling in head-and-neck squamous cell carcinoma (HNSCC), we observed similar alterations of ECM proteins induced by Activin A stimulation, which could no longer be deteted upon utilizing knockout of ACVRIB in the HNSCC lines (data not shown).

Taken together, we show that in contrast to the dysplastic ECdnT cells and the TE-2 ESCC cell line, fibroblast-secreted Activin A could not suppress TE-11 cell invasion nor were ECM components altered, indicating the necessity of intact Activin A signaling to mediate these effects. In the absence of ACVRIB, we presume the tumor cells can escape Activin A-meditated regulation with reciprocal consequences on the ECM itself. This would explain the observation of increased tumor stage in the human tissue set for which stromal Activin A was high in tumors with low ACVRIB expression.

### Angiogenesis assessed by endothelial tube formation is significantly inhibited following treatment with conditioned media from Activin A overexpression cultures

**Fig. 6:**
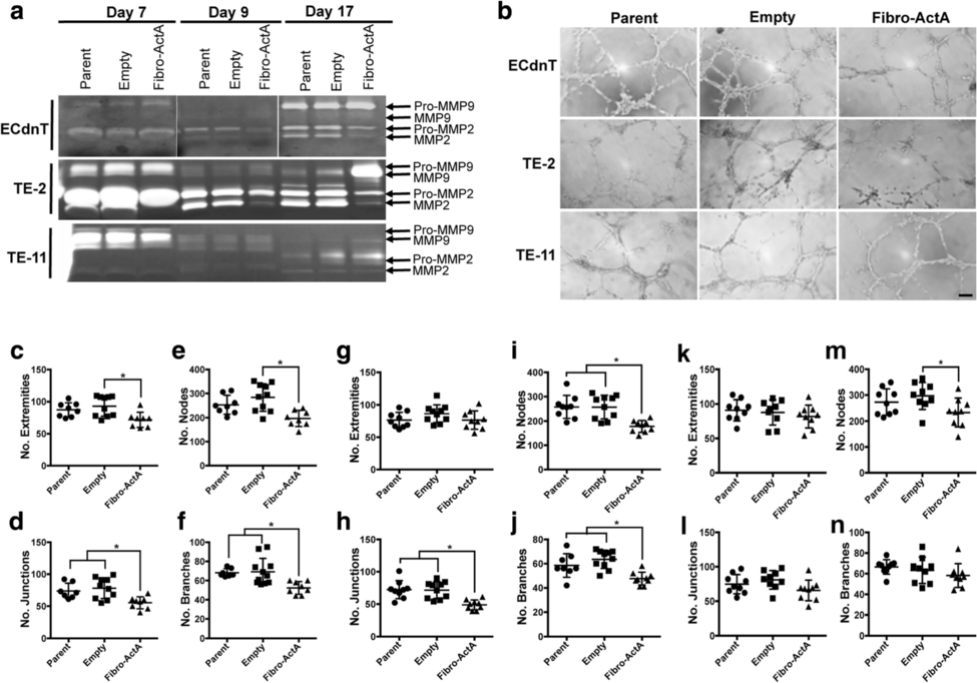
Fibro-ActA conditioned media from three-dimensional organotypic cultures inhibits *in vitro* angiogenesis. **a** Gelatin zymography of conditioned media collected from 3D-organotypic cultures at day 7 (fibroblasts alone), day 9 (media collection following the addition of epithelial cells), and day 17 (final collection). Arrows indicate the locations of pro- and cleaved MMP-2 and MMP-9. **b** Representative images of endothelial tube formation assays using HMEC-1 cells. HMEC-1 endothelial cells cultured with day 9 conditioned media from ECdnT/Fibro-ActA and TE-2/Fibro-ActA cultures had less overall tube formation, compared to parent and empty vector controls. This effect was not observed upon treatment with TE-11/Fibro-ActA conditioned media. Quantification of endothelial extremities, nodes, junctions, and branches for the assays with ECdnT (**c**-**f**), TE-2 (**g**-**j**), and TE-11 (**k**-**n**) conditioned media shown in (**b**)

Because matrix metalloproteases are known to be regulated by TGFβ signaling pathways and have long been implicated as necessities for epithelial cell invasion, we performed gelatin zymography to examine total and active MMP-2 and MMP-9 expression. We found that expression of pro- and cleaved MMP-2 were reduced in Fibro-ActA cultures from ECdnT and TE-2 cells, yet their expression in TE-11 was not affected by fibroblast overexpression of Activin A throughout the 17-day culture (Fig. 6a). Overall, MMP-9 expression and activity remained largely unchanged in the different culture conditions, aside from an increase in active MMP-9 in the TE-2 cells at the end of the culture (day 17) in the presence of Fibro-ActA. These results suggest that, even in the presence of MMPs, which promote epithelial cell invasion, Activin A is able to suppress this effect.

As MMPs, particularly MMP-2 and MMP-9, are necessary for tumor angiogenesis (reviewed in [55]) and Activin A has been characterized as a potent inhibitor of this process [14, 56], we next examined the effect of Activin A overexpressing cultures on endothelial tube formation. We found that treatment of HMEC-1 endothelial cells with conditioned media from dysplastic ECdnT cultured with Fibro-ActA showed reduced tube formation, compared to parent and empty vector control conditioned media (Fig. 6b). This effect was also observed when using TE-2 conditioned media from Fibro-ActA (Fig. 6b). Quantitative analysis of the assay images showed that HMEC-1 cells treated with Fibro-ActA conditioned media from ECdnT and TE-2 cultures formed significantly less extremities, nodes, junctions, and branches, compared to parent and empty vector control (Fig. 6c-j). These results indicate that overexpression of Activin A, and its crosstalk with the epithelial cell compartment in this context, results in the inhibition vessel formation, ultimately leading to suppression of tumor cell expansion. Conditioned media from TE-11 cells cultured with Fibro-ActA, however, failed to reduce tube formation and only a significant reduction of nodes was observed (Fig. 6b, k-n). Interestingly, treatment of HMEC-1 cells with recombinant protein (TGFβ, Activin A, or the Activin A antagonist Follistatin) or A83-01 were equally effective at inducing tube formation (Additional file 4: Figure S4a-e). These results indicate that Activin A alone may not directly inhibit angiogenesis, however it may regulate the expression of several anti-angiogenic proteins to control this process through epithelial-mesenchymal crosstalk.

## Discussion

It has been well documented that TGFβ receptors, which are closely related to Activin receptors, are downregulated as a means to evade growth suppression. In colorectal cancer, aside from loss of Smad4, TβRI and TβRII are commonly downregulated or lost [57]. Similar observations have been made in laryngeal [58] and gastric cancers (reviewed in [59]). The downregulation of these receptors occurs concurrently with TGFβ upregulation [57]. As there are significant similarities between these two pathways, the analogous observations made between the TGFβ pathway, as shown in the literature, and the Activin A pathway, demonstrated here, are not surprising. *ACVR2* mutations have been described to attenuate Activin A signaling in prostate cancer [60] and microsatellite unstable colon cancer [61]. The mutations identified are similar to the well-characterized frameshift mutations in *TGFBR2* [62]. Inactivation of ACVRIB so far has only been identified in pancreatic cancer as a consequence of a somatic mutation [63], and homologous deletion is associated with an aggressive phenotype in pancreatic cancer [64]. Based on the 120 esophageal squamous tumor tissues we analyzed in this study, we found that ACVRIB can also be lost in ESCC. Furthermore, while ACVRIB loss has not been described to contribute to esophageal cancer, the overexpression of Activin A has been identified previously to be associated with enhanced matrix metalloprotease expression [26] and ESCC aggressiveness [65], partially through upregulated of N-cadherin [24]. We have additionally addressed the loss of ACVRIB in the context of head and heck squamous cell carcinoma and have observed results similar to the data presented here (data not shown).

In our study we found not only that Activin A is increased, but also that its location to the stroma is of importance to the function. When overexpressed in the tumor, Activin A confers differential effects. Some cancers, such as lung and head and neck squamous cell carcinoma, develop insensitivity to the growth inhibitory effects of Activin A, one of the hallmarks of cancer [66–69]. In prior studies, we have described that treatment of premalignant cultures recombinant Activin A induces esophageal cell invasion [34]. In this context, however, signaling is not directional, as both epithelial and stromal compartment are exposed to the recombinant Activin A. In the current study, we mimic the stromal source of Activin A, which we observed in the patient tissues. The fibroblast-secreted Activin A therefore contrasts stimulation of the whole culture with recombinant protein Activin A, showing inhibition of esophageal cell invasion. This inhibition is a consequence of intact Activin A signaling; however, loss of ACVRIB allows esophageal cancer cells, such as TE-11 cells, to escape Activin A-dependent regulation.

Though the results of our study provide significant insight into the role of Activin A signaling in cancer progression, limitations remain. The three-dimensional organotypic reconstruct culture system is an efficient way to examine cellular crosstalk *in vitro*, however it is a simplified environment and does not account for additional components of the microenvironment, such as endothelial cells or leukocytes. Activin A may be secreted by, and signal within, a variety of cell types, including those listed. In our study, we used fibroblasts as a source of stromal Activin A, therefore accounting for Activin A expression alone, but not effects within the assorted cell types. The integration of different cell types into this system will provide further into the systemic effects of Activin A during cancer progression. We aimed to focus on angiogenesis as one example of the complex microenvironment, and to identify the functional consequences of fibroblast-Activin A overexpression using the endothelial tube formation assay. Activin A has been consistently shown to function as a potent anti-angiogenic factor, contrary to reports indicating its oncogenic role. Treatment of endothelial cells showed reduced tube formation possibly through inhibition of proliferation [4, 14, 70–79]. In this study, we describe a reduction of tube formation, as measured by the number of nodes, junctions and branches, dependent on intact Activin A signaling. Additionally, we observed that fibroblast-derived overexpression of Activin A downregulates vascular endothelial growth factor expression, one of the key components of tumor angiogenesis (data not shown). Previous research identified that the majority of endothelial cells expresses ActRII/IIB and are therefore able to respond to Activin A ligand binding [66, 72, 78–83]. However, as we use conditioned media from fibroblast-secreted Activin A organotypic cultures, the effects we measured on tube formation may be indirect. *In vitro* co-culture studies of fibroblasts and endothelial cells in the presence of ESCC cells have shown that tumor cell-secreted TGFβ can activate fibroblasts resulting in VEGF secretion and increased formation of endothelial network formation [84]. A number of pro-angiogenic factors were downregulated when we performed angiokine arrays (data not shown), which may be under the regulation of Activin A. Additionally, Activin A has been found to regulate several secreted factors, such as IL-8, VEGF, GnRH, and PTGS2 [85–87]. Therefore, it can be difficult to conclude that Activin A overexpression alone is responsible for all of the observed effects in our experiments, particularly since contradictory evidence has been previously observed [34]. However, our study investigates a long-term stable overexpression of Activin A in a three-dimensional context, rather than the utilization of recombinant Activin A for *in vitro* treatments. Therefore, in this study, we are able to investigate the paracrine signaling effects due to epithelial-fibroblast crosstalk.

In this paper, we showed that Activin A plays a necessary role in controlling mechanisms of esophageal squamous cell carcinoma invasion and, most likely, tumor progression. Activin A signaling from the stroma, e.g. fibroblasts, regulates expression of extracellular matrix proteins and thereby the signaling pathways involved in cell invasion. It has been found the expression of Activin type I receptors (ActRI and ACVRIB) and type II receptor (ActRII) mediates Activin A effects in collagen gel contraction using human lung fibroblasts [88]. In pancreatic stellate cells, autocrine Activin signaling induces activation and collagen secretion [89]. We observed the loss of podoplanin and fibronectin in response to overexpression of Activin A, but also αSMA- and podoplanin-double positive fibroblasts in most of the invasive cultures. In the context of fibrosis, which is characterized by the presence of myofibroblasts, Activin A has been shown to regulate myofibroblast differentiation mediated by integrin α11β1, a collagen receptor on fibroblasts [90].

Interestingly, we believe that Activin A, when unable to bind and signal through ACVRIB, is then free to associate with other receptor complexes, such as BMPR2 or BMPR1/ALK2, and propagate a signal [91, 92]. This data highlights, for the first time, the importance of maintaining an intact Activin A signaling pathway to control esophageal squamous cell carcinoma invasion. Previous literature has shown that Activin A can bind with low affinity and transduce a signal through subsequent TGFβ family receptors, of particular note the BMP receptors [93]. Substantial research has demonstrated that induction of the BMP pathway can result in production of collagens, commonly leading to bone formation [94, 95]. In fact, Activin A has been reported to regulate ECM mineralization, as well as enhance BMP-induced formation of bone and collagen [96, 97]. This may be a mechanism to control ECM formation aside of ACVRIB-dependent Activin A signaling.

## Conclusion

In conclusion, we have demonstrated that in a dysplastic esophageal squamous microenvironment, Activin A works to inhibit cell invasion into the stroma, however, when the pathway becomes dysregulated, such as through the downregulation of ACVRIB, cells are rendered unresponsive. Dysregulation of the Activin A pathway may be a way in which cancer cells can adapt to circumvent inhibitory environmental factors.

## Additional files

Additional file 1: Figure S1. “Epithelial and stromal markers do not vary between ESCC patient samples, by stage”; control staining and analysis of TMA data. A comparison of ESCC patient samples, using immunofluorescence staining on a prepared microarray, showed that epithelial keratin 14 (K14) (a), stromal vimentin (b), the extracellular matrix protein collagen (c), and monocyte marker CD68 (d) expression did not significantly differ between stage and esophageal squamous cell carcinoma (SCC) versus lymph node metastasis (LN Met). (TIF 4401 kb)
Additional file 2: Figure S2. “Overexpression of Activin A, validated by ELISA, was persistent and did not affect fibroblast contractility”; validation of assays by ELISA and cell contractility assay. (a) Overexpression of Activin A was validated following each retrovirus transduction. Levels of secreted Activin A protein, measured in conditioned media, were significantly higher in Act A compared to parent and empty vector control, ECdnT, TE-2, and TE-11 cells in 2D monolayer. Overexpression of Activin A was validated throughout the 17-day organotypic culture with ECdnT (b), TE-2 (c), and TE-11 (d). Fibro-ActA had sustained increased expression of Activin A during this time period. (e) Parent, empty, and Fibro-ActA had comparable ability to contract collagen, indicating that overexpression of Activin A alone did not hinder the fibroblasts ability to contract the extracellular matrix. 3-butanedione monoxime (BDM) was used as an assay control. (f) Quantification (percent) of fibroblast matrix contraction from (e). (One-way ANOVA, *p<0.05). (TIF 4375 kb)
Additional file 3: Figure S3. “Activin A overexpression reduced proliferation of TE-2, but not ECdnT and TE-11 cells”; quantification of Ki67 from ECdnT, TE-2, and TE-11 organotypic cultures in Figs. 2, 4, and 5. Proliferation of (a) ECdnT, (b) TE-2, and (c) TE-11 esophageal cells, as measured by nuclear Ki67 immunofluorescence staining, in three-dimensional organotypic cultures with parent, empty, and Fibro-ActA. (One-way ANOVA, *p<0.05). (TIF 4401 kb)
Additional file 4: Figure S4. “Endothelial tube formation assays following treatment with recombinant proteins and the chemical inhibitor, A83-01”; endothelial tube formation assays and quantification following treatment. (a) Brightfield images of HMEC-1 endothelial tube formation assays treated with recombinant protein (TGFb, Activin A, or Follistatin) or the chemical inhibitor A83-01. (b) Treatment of HMEC-1 cells with Activin A, Follistatin, or A83-01, but not TGFb, increased the number of formed endothelial extremities. (c-d) Compared to media control, treatment with recombinant protein (TGFb, Activin A, Follistatin) or A83-01 increased the number of endothelial tube nodes and junctions, respectively. (e) Treatment of HMEC-1 cells with Activin A, Follistatin, or A83-01, but not TGFb, increased the number of formed endothelial extremities. (One-way ANOVA, *p<0.05; scale bar = 200 µm). (TIF 4401 kb)

## Abbreviations

ACVRIB: Activin receptor IB
ACVRIIB: Activin receptor IIB
BMP: Bone morphogenetic protein
EAC: Esophageal adenocarcinoma
ECdnT: E-cadherin/TβRII dominant-negative
ECM: Extracellular matrix
ESCC: Esophageal squamous cell carcinoma
TGFβ: Transforming growth factor β
