# Supplementary figures and images for: Esophageal squamous cell carcinoma invasion is inhibited by Activin A in ACVRIB-positive cells
Source: BMC Cancer. 2016 Nov 9;16:873. doi: 10.1186/s12885-016-2920-y (PMC5101642; doi:10.1186/s12885-016-2920-y)

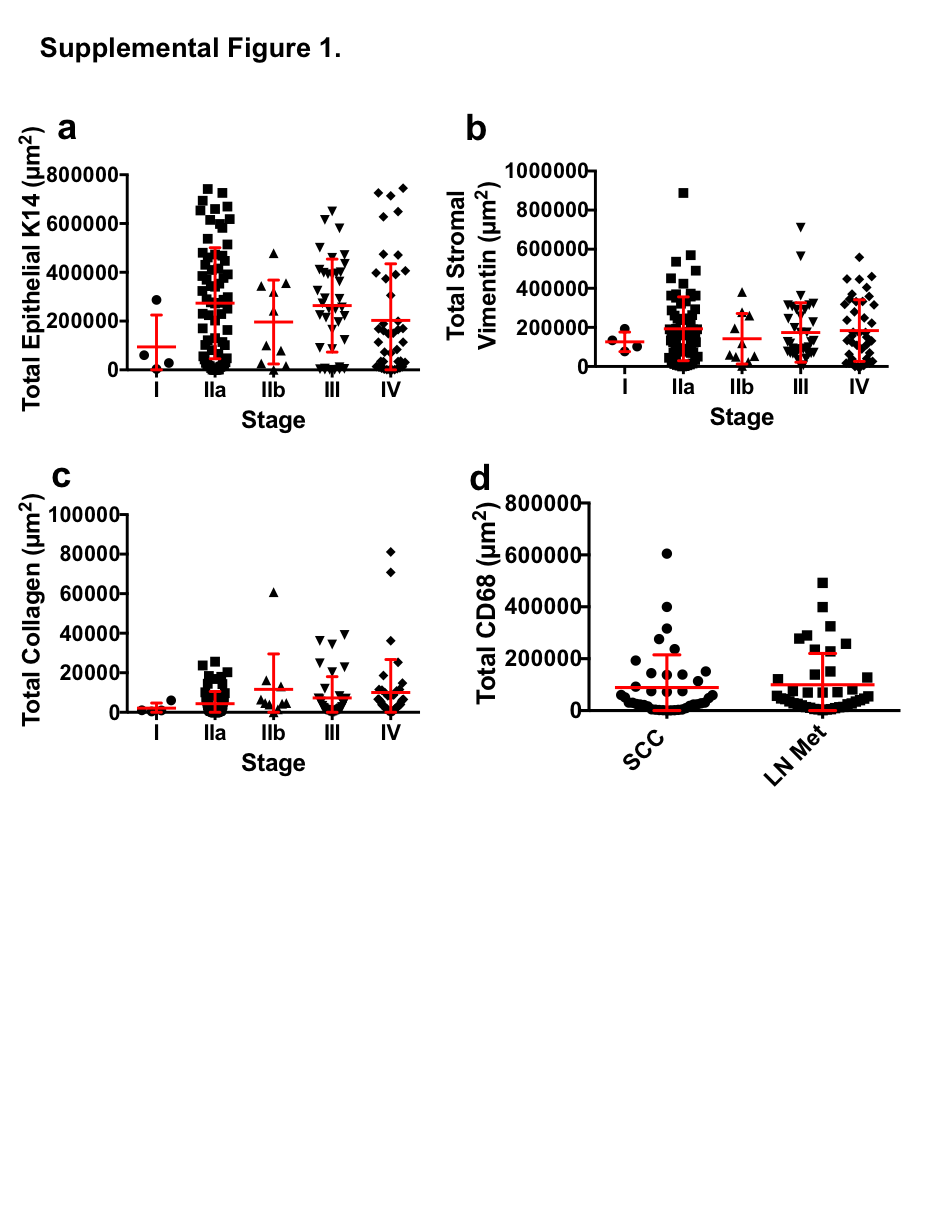

Supplement: Additional file 1: Figure S1. — “Epithelial and stromal markers do not vary between ESCC patient samples, by stage”; control staining and analysis of TMA data. A comparison of ESCC patient samples, using immunofluorescence staining on a prepared microarray, showed that epithelial keratin 14 (K14) (a), stromal vimentin (b), the extracellular matrix protein collagen (c), and monocyte marker CD68 (d) expression did not significantly differ between stage and esophageal squamous cell carcinoma (SCC) versus lymph node metastasis (LN Met). (TIF 4401 kb) [file 12885_2016_2920_MOESM1_ESM.tif]

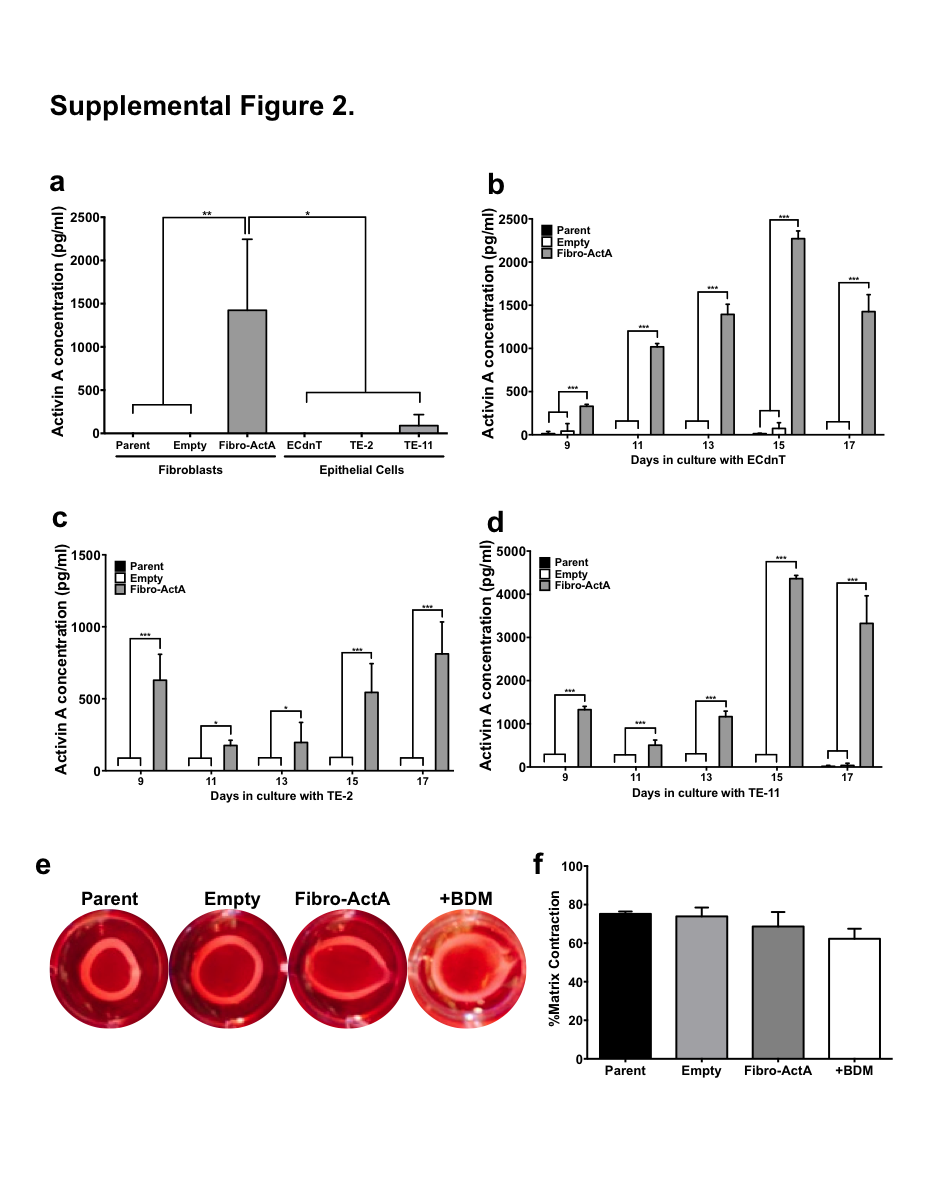

Supplement: Additional file 2: Figure S2. — “Overexpression of Activin A, validated by ELISA, was persistent and did not affect fibroblast contractility”; validation of assays by ELISA and cell contractility assay. (a) Overexpression of Activin A was validated following each retrovirus transduction. Levels of secreted Activin A protein, measured in conditioned media, were significantly higher in Act A compared to parent and empty vector control, ECdnT, TE-2, and TE-11 cells in 2D monolayer. Overexpression of Activin A was validated throughout the 17-day organotypic culture with ECdnT (b), TE-2 (c), and TE-11 (d). Fibro-ActA had sustained increased expression of Activin A during this time period. (e) Parent, empty, and Fibro-ActA had comparable ability to contract collagen, indicating that overexpression of Activin A alone did not hinder the fibroblasts ability to contract the extracellular matrix. 3-butanedione monoxime (BDM) was used as an assay control. (f) Quantification (percent) of fibroblast matrix contraction from (e). (One-way ANOVA, *p<0.05). (TIF 4375 kb) [file 12885_2016_2920_MOESM2_ESM.tif]

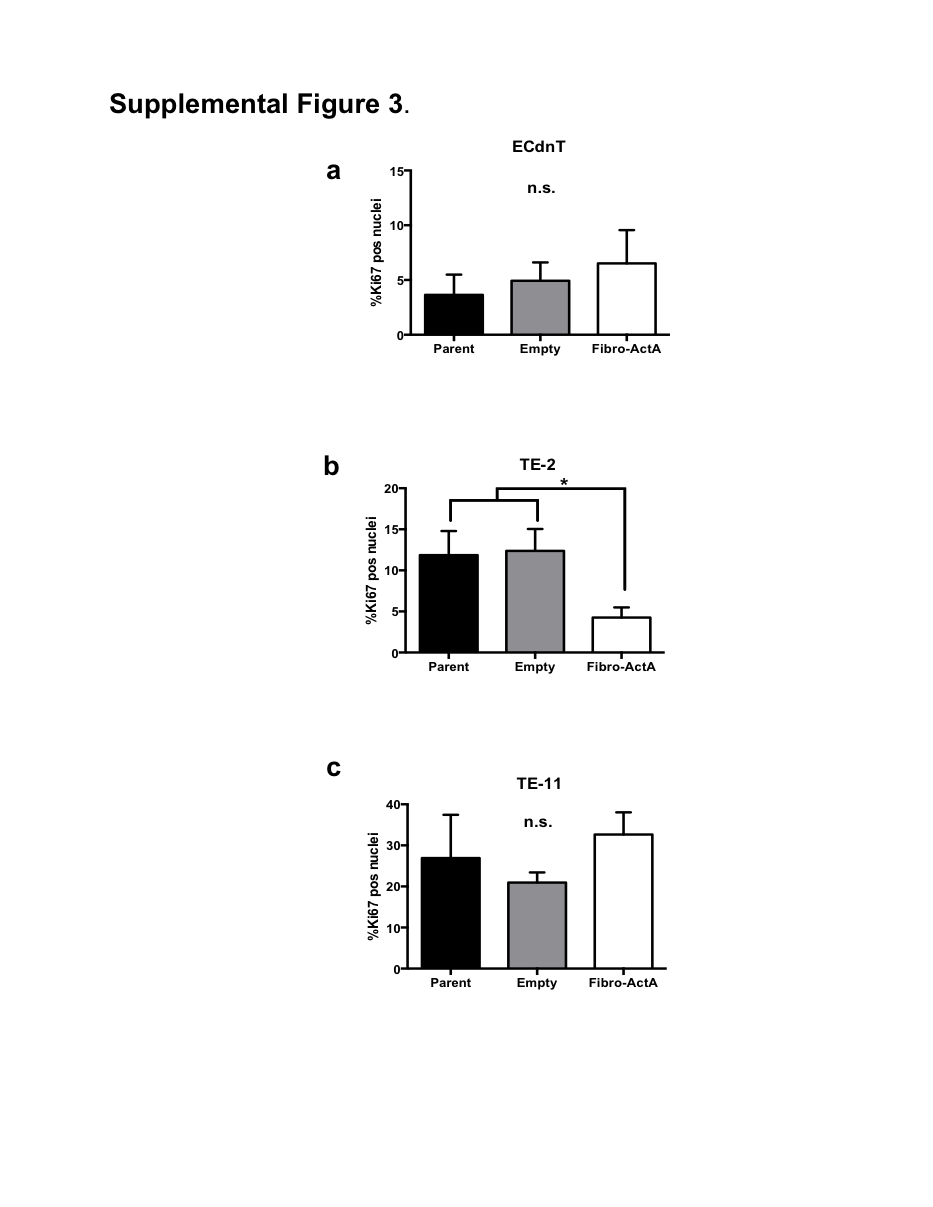

Supplement: Additional file 3: Figure S3. — “Activin A overexpression reduced proliferation of TE-2, but not ECdnT and TE-11 cells”; quantification of Ki67 from ECdnT, TE-2, and TE-11 organotypic cultures in Figs. 2, 4, and 5. Proliferation of (a) ECdnT, (b) TE-2, and (c) TE-11 esophageal cells, as measured by nuclear Ki67 immunofluorescence staining, in three-dimensional organotypic cultures with parent, empty, and Fibro-ActA. (One-way ANOVA, *p<0.05). (TIF 4401 kb) [file 12885_2016_2920_MOESM3_ESM.tif]

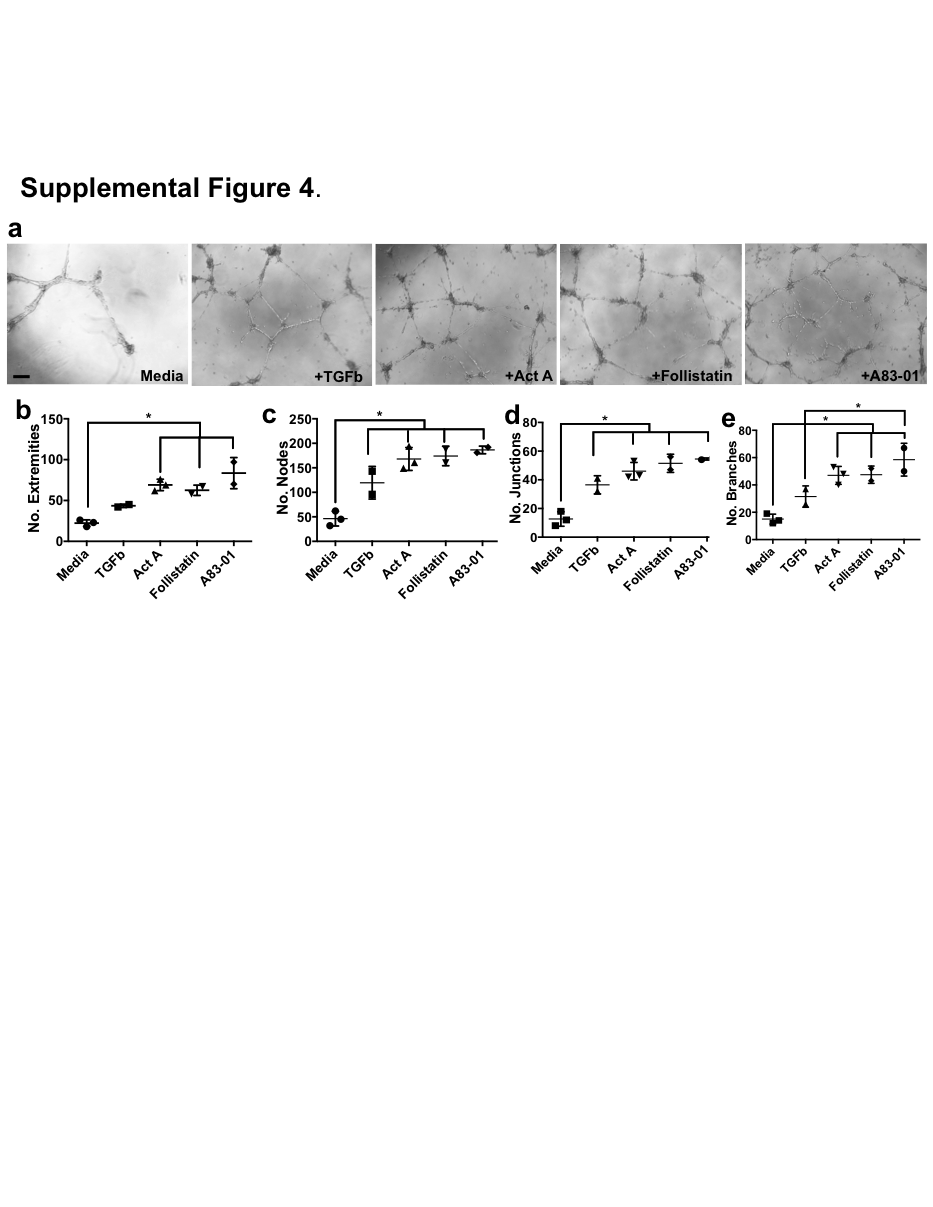

Supplement: Additional file 4: Figure S4. — “Endothelial tube formation assays following treatment with recombinant proteins and the chemical inhibitor, A83-01”; endothelial tube formation assays and quantification following treatment. (a) Brightfield images of HMEC-1 endothelial tube formation assays treated with recombinant protein (TGFb, Activin A, or Follistatin) or the chemical inhibitor A83-01. (b) Treatment of HMEC-1 cells with Activin A, Follistatin, or A83-01, but not TGFb, increased the number of formed endothelial extremities. (c-d) Compared to media control, treatment with recombinant protein (TGFb, Activin A, Follistatin) or A83-01 increased the number of endothelial tube nodes and junctions, respectively. (e) Treatment of HMEC-1 cells with Activin A, Follistatin, or A83-01, but not TGFb, increased the number of formed endothelial extremities. (One-way ANOVA, *p<0.05; scale bar = 200 µm). (TIF 4401 kb) [file 12885_2016_2920_MOESM4_ESM.tif]
